# Enabling teleophthalmology during the COVID-19 pandemic in the Province of Trento, Italy: Design and implementation of a mHealth solution

**DOI:** 10.1371/journal.pone.0257250

**Published:** 2021-09-10

**Authors:** Giulia Malfatti, Elisabetta Racano, Roberta Delle Site, Lorenzo Gios, Stefano Micocci, Marco Dianti, Pietro Benedetto Molini, Francesca Allegrini, Mariangela Ravagni, Monica Moz, Andrea Nicolini, Federica Romanelli

**Affiliations:** 1 TrentinoSalute4.0, Competence Center for Digital Health of the Province of Trento, Trento, Italy; 2 Azienda Provinciale per i Servizi Sanitari, U.O. di Oculistica, Ospedale di Rovereto, Trento, Italy; 3 Fondazione Bruno Kessler, Digital Health Lab, Povo, Italy; Universita degli Studi della Campania Luigi Vanvitelli, ITALY

## Abstract

**Background:**

Due to the many restrictions imposed during the COVID-19 emergency, the normal clinical activities have been stopped abruptly in view of limiting the circulation of the virus. The extraordinary containment measures have had a dramatic impact on the undertaking and follow-up of ophthalmic outpatients.

**Objective:**

In order to guarantee proper monitoring and routine care, the Pediatric Ophthalmology equipe of Rovereto Hospital (North-East of Italy) supported by the Competence Center on Digital Health TrentinoSalute4.0, designed and implemented a digital platform, TreC Oculistica, enabling teleophthalmology. We report our innovative—albeit restricted—experience aiming at testing and maximizing the efficacy of remote ophthalmic and orthoptic visits.

**Methods:**

A multidisciplinary team created the TreC Oculistica platform and defined a teleophthalmology protocol. The system consists of a clinician web interface and a patient mobile application. Clinicians can prescribe outpatients with the App and some preliminary measurements to be self-collected before the televisit. The App conveys the clinician’s requests (i.e. measurements) and eases the share of the collected information in a secure digital environment, promoting a new health care workflow.

**Results:**

Four clinicians took part in the testing phase (2 ophthalmologists and 2 orthoptists) and recruited 37 patients (mostly pediatric) in 3 months. Thanks to a continuous feedback between the testing and the technical implementation, it has been possible to identify pros and cons of the implemented functionalities, considering possible improvements. Digital solutions such as TreC Oculistica advance the digitalization of the Italian health care system, promoting a structured and effective reorganization of the workload supported by digital systems.

**Conclusions:**

The study tested an innovative digital solution in the teleophthalmology context and represented the first experience within the Italian healthcare system. This solution opens up new possibilities and scenarios that can be effective not only during the pandemic, but also in the traditional management of public health services.

## Introduction

In December 2019, the first cases of Coronavirus disease (COVID-19) emerged as a consequence of SARS-CoV-2 virus. COVID-19 is a contagious and potentially lethal respiratory infection that has resulted in a global pandemic with devastating consequences both for the economy and the healthcare systems worldwide [1]. Countries have worked to stem the spread of the infection using social distancing and stay-at-home orders [2, 3], whilst healthcare systems around the world have been forced to rapidly integrate telehealth and digital innovations to mitigate the risk of virus transmission. As a result, what started as a necessity is now widely regarded as the “new normal”.

Starting from March 2020, the American Academy of Ophthalmology (AAO) published updated recommendations for reducing the risk of SARS-CoV-2 transmission in clinics, including a questionnaire to identify patients with potential exposure to SARS-CoV-2, protocols for scheduling and visiting patients, environmental cleaning and disinfection recommendations, as well as guidelines about managing elective ophthalmic care, including and encouraging the use of telehealth services [4].

Teleophthalmology enables clinicians to evaluate at a distance patients’ ocular diseases. In principle, this approach can have a positive impact as it can facilitate a more efficient and equitable distribution of often limited healthcare resources compared to the standard healthcare delivery. In particular, during the outbreak of COVID-19, telemedicine allowed triaging patients even before their access to the medical facilities, avoided unnecessary visits by monitoring and performing remote visits, and thus reduced infection risks in the healthcare setting [5]. Innovative approaches combining video-consultations and digital services, such as remote instrumental examination, have already been adopted in clinical settings, showing an improvement in the quality of telemedicine consultations while allowing patients to access clinical services from their homes and remote areas [6–8]. Nowadays, the majority of teleophthalmology services adopts asynchronous (i.e. store-and-forward of images and measurements) or hybrid (i.e. real-time and asynchronous approaches) communication methods, while only few rely on synchronous methods only (i.e. video conferencing) [9, 10].

Ophthalmology can rely on several techniques aimed at screening, following up and treating patients with different ocular diseases, techniques that can be performed also at a distance, typically by trained staff. In this context, smartphones play a pivotal role in the teleophthalmology field [11]: they allow performing specific ophthalmic medical tests (e.g. visual acuity measurement), acquiring high resolution images of the eye and sharing them with the ophthalmologist for diagnosis and management (mHealth). Smartphones use is widely spread even in rural and remote areas around the world, promoting and improving the access to medical services [12, 13]. Mobile technologies also provided a new framework for digital connectivity of ophthalmic diagnostic devices, supporting real time decision-making and streamlining diagnostic processes for eye-care professionals [14].

In recent years, far before the COVID-19 pandemic, the number of ophthalmic mobile applications increased significantly. The technological development contributed in changing the traditional patient-doctor relationship by introducing remote-testing methods and encouraging patients to monitor their health conditions and supporting clinicians in the assessment phase. Nevertheless, before the COVID-19 emergency, these innovations did not affect all the ophthalmic subspecialties equally, and were not diffusely adopted within traditional healthcare systems [11]. The pandemic triggered the need for investigating alternative approaches in providing ophthalmic care. For example, the Child Sight Institute of Hyderabad in India, in a retrospective analysis, concludes that teleconsultation was extremely useful in diagnosing and monitoring pediatric ophthalmology and strabismus during COVID-19 emergency [15]. In the Province of Pescara (Italy) the ophthalmology department developed a specific protocol to manage outpatients and follow-ups. They activated a dedicated telephone number, so that patients that called were first administered with a questionnaire that allowed the specialist to decide the best therapy or to schedule a visit in the office [16]. The remote assessment of visual function has been tried even before the pandemic [17, 18], nevertheless, it was limited by the number of items that could be presented and by the means used to administrate questionnaires, mainly telephonically. Nevertheless, in spite of encouraging examples of tele-consultation in ophthalmology [19], the use of innovative tools is still a scattered phenomenon, whilst in Italy telemedicine has not been developed nor adapted for ophthalmology so far.

In this paper we describe an innovative and, to the best of our knowledge, unique experience conducted in 2020 in the Province of Trento (North-East of Italy). The group of pediatric ophthalmology of Rovereto Hospital, together with the multidisciplinary group of the Competence Center on Digital Health TrentinoSalute4.0, designed and implemented an organizational protocol for managing outpatients, follow-ups and screenings. The protocol was enabled by a new technological system purposely designed to promote teleophthalmology during the COVID-19 outbreak. The system consists of a clinical digital platform and a smartphone-based App, *TreC Oculistica*. The core pillar of this innovative organizational model lies in the new role that is assigned to the patient and the new doctor-patient relationship that is promoted through this initiative. Thanks to the technological assets and tools available, a hybrid model is adopted, where patient-collected and provider-collected pieces of information are used together to support healthcare staff in taking informed decisions and monitoring the eyesight conditions of outpatients.

We will provide a description of the system and its functioning, emphasizing the organizational protocol adopted to test the technological asset. We will then present the experience performed with a small sample of patients. Finally, we will discuss the overall initiative, pointing at pitfalls we have encountered and at lessons learned.

## Materials and methods

The system, TreC Oculistica, was named by merging the patient’s medical record digital platform used in the Trentino Italian Region (TreC) [20] and the ophthalmology clinical field (Oculistica). TreC Oculistica was designed to allow a structured remote interaction between patients and healthcare staff, and to facilitate a new workflow organization. It consists of a web based clinical dashboard, a functional layer (backend) implementing the business logic of the platform, used by the healthcare practitioner, and a mobile application that patients can download free of charge on their smartphone and that clinicians need to activate and prescribe to each patient. The reason why we chose to realize a mobile App is to reduce possible inequities in the access to televisit services, since smartphone use is widely spread within the community, regardless of the socio-economic status of the users. In fact, the 2019 *Global mobile market report* shows that 60.8% of Italian citizens actively use a smartphone, and that this percentage is likely to increase through the years [21].

**Fig 1:**
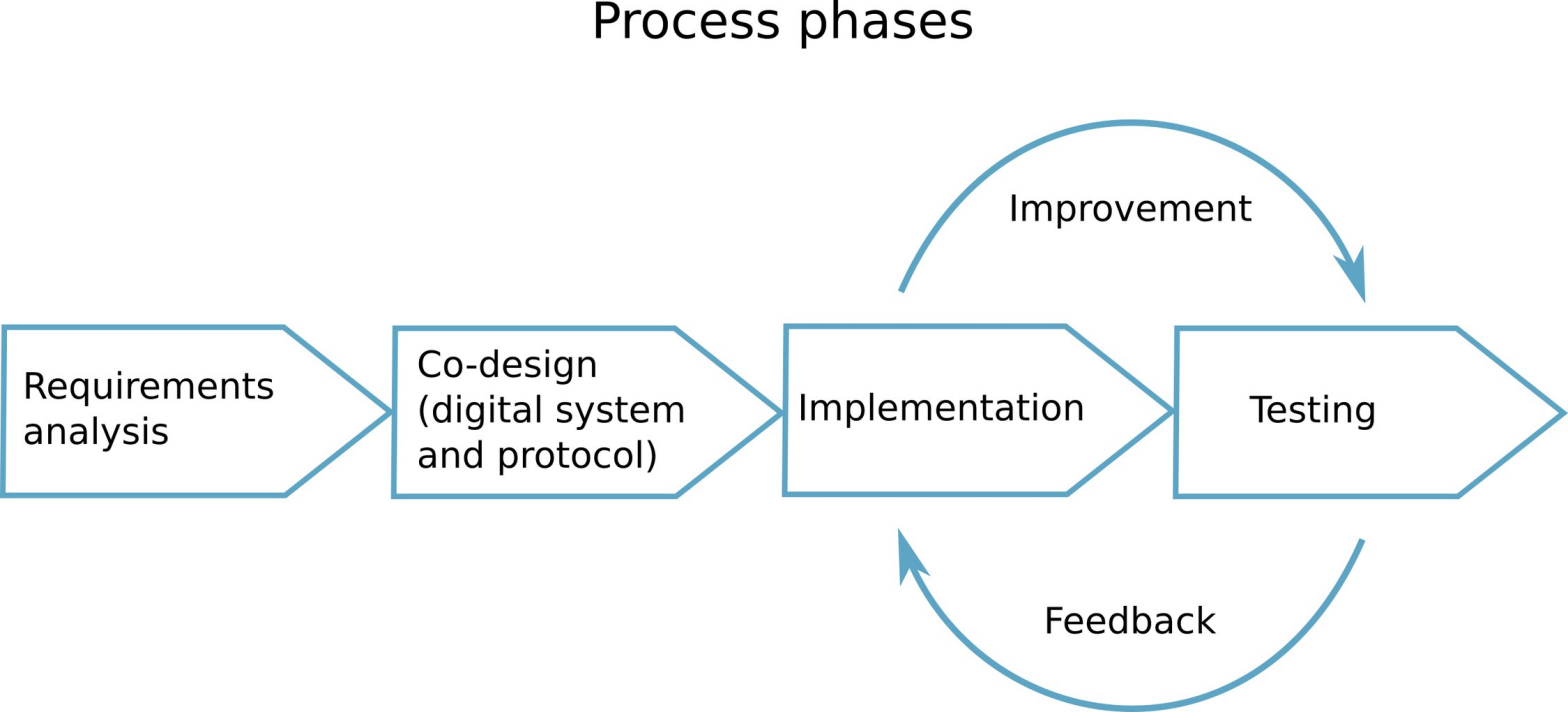
Process phases. a sequential approach was adopted. The first phase consisted in the *requirements analysis* followed by the *co-design* of the technological solution and definition of the operative protocol for teleophthalmology. The third phase comprised the *implementation* of the digital solution (TreC Oculistica system) and the fourth phase the *testing* of the system and protocol. A continuous exchange of feedback between the implementation and testing phases allowed to identify and implement possible improvements.

To develop TreC Oculistica system, we adopted a structured and sequential approach (Fig 1) aimed at building a technology that (i) empower the patient as active stakeholder in the process, (ii) help and support healthcare practitioners in taking informed decisions and providing effective healthcare services for outpatients, and (iii) convey a different workload organization by simplifying the management of patients at a distance and allowing proper follow-up, whilst reducing patients’ attendance to the Pediatric Ophthalmology clinic.

The initiative was approved by the Provincial Healthcare Service in line with the healthcare strategy promoted by the Province of Trento as political stakeholder. According to Italian legislation, neither approval from the ethics committee nor informed consent from the participants is required in case both scope and purpose of data processing are part of the provision of healthcare service. Only MD and clinical staff had access to patient’s data for clinical purposes only. Patient’s data included in the manuscript were obtained in a fully anonymized and de-identified manner.

### Organizational response

In the very first months of the outbreak, the Ophthalmic Unit of Rovereto Hospital was forced to suspend all the outpatient’s services, limiting the clinic access to emergency visits only. This abrupt interruption led to bewilderment among patients and to logistic difficulties in providing outpatients healthcare, due to (i) the reduced visits space, (ii) the increased sanitation procedures and (iii) the need for proper social distancing. Within a few weeks, from March to April 2020, the number of non-delivered health care services increased dramatically, exceeding 2000 units.

To deal with this unprecedented situation, affecting not only the Operative Unit of Ophthalmology but most of the healthcare branches, the Autonomous Province of Trento (PAT) and the Provincial Healthcare Service (APSS) applied specific actions, namely:

- Updating the nomenclator of outpatient specialist assistance services by formally including telemedicine among the offered services [22].
- Adapting the national guidelines to the specific regional context, indicating and clarifying the procedure for healthcare staff conducting and accounting a televisit. During the pandemic, all the telemedicine services have been provided on a co-pay-exempt basis, promoting equity and accessibility to telemedicine healthcare services.

### Ophthalmology unit organization

Even though ophthalmology strongly relies on instrumental examinations, there are yet some clinical measurements that can be acquired without the need of any professional equipment, allowing the evaluations of a relevant number of eyesight conditions by means of patient-collected information. As reported in the introduction, the ability to provide eye care services from a distance is already a standard in some countries (e.g. USA, Canada, Australia, India). Looking at those good practices, we tested an innovative approach to telemedicine. Easy to use printable materials and digital mobile applications were purposely selected by the healthcare staff (see paragraph 1.5) and patients were invited to actively contribute to the evaluation of their eyesight condition by providing healthcare staff with some specific parameters collected autonomously using the suggested materials (patient-collected information).

The protocol adopted in the first months of pandemic consisted in contacting the already booked-for-a-visit patient telephonically, offering the possibility to undergo a remote visit. Instructions and materials were then sent by email seven to ten days before the scheduled televisit together with another email with the link to the video-call platform (i.e.Google Meet). Patient’s measurements were collected via email and evaluated before the televisit. After the televisit, the healthcare practitioner produced a medical report.

This initial organization, adopted at the very peak of the pandemic, was long and complex, requiring a considerable amount of time and energy for the healthcare staff to collect and organize the material sent and received before the televisit. Moreover, the digital platform used to exchange sensible clinical information and to perform televisit could not guarantee the appropriate privacy and security levels in data sharing. Thanks to the support of the Competence Center on Digital Health TrentinoSalute4.0, a multidisciplinary group including medical doctors, project managers, informatics and technologists, designed and implemented the technological solution for teleophthalmology TreC Oculistica, simplifying the organization of the activity both for healthcare practitioners and for patients.

In the following paragraphs we report the stages that led to the creation of the system.

### Requirements

The first task of the working group was to define the requirements of the system.

As stated before, the necessity was to simplify the steps to be conducted prior to the televisit (i.e. material delivery, information collection, televisit execution) by providing patients with appropriate indications and materials, and by organizing the reception of preliminary measurements required to avoid missing data. This led the working group to design (i) an easy-to-use mobile App for patients and (ii) a dedicated web interface for healthcare staff.

Patient’s App main functionalities:

- Video-call: a secure and encrypted channel that allows the safe execution of the televisit without relying on third party services;
- Chat: an asynchronous encrypted communication channel to be used in the period before the visit, to exchange additional information with the clinician and to clarify possible doubts;
- Televisit preparation area: a section purposely organized to guide patients in getting ready for the televisit. Patients can find information about the measurements they are asked to acquire autonomously and in which order is needed to insert the collected data;
- Tutorials area: a list of detailed instructions on how to perform each requested measurement;
- Measurements archive: a section where data inserted for previous televisit appointments are stored;

Complementarily, the functionalities identified for the clinical web interface are:

- Patient’s profile and App activation and prescription function: clinicians have to activate patients’ profiles in the clinician web interface in order to be able to prescribe the use of the App, allowing patients to access the App functionalities.
- Video-call: that allows programming and executing televisits;
- Chat: that the clinician can activate or deactivate depending on the need, through which providing assistance during the collection of measurements;
- Checklist of measurements: a list of all the materials and measurements integrated in the TreC Oculistica App that can be provided to patients. The measurements selection can be personalized patient by patient, selecting those more appropriate from the checklist;
- Dashboard: a dedicated area for each patient where all the information shared with and collected from the patient through time are grouped and organized and can be consulted before the televisit. Through the dashboard, the clinician can access the list of all the televisits performed with a specific patient and compare the data collected with those of previous televisits.

### System development

#### The technological substrate: TreC digital platform

TreC Oculistica is a digital platform specialized on an already existing digital platform named TreC (*Cartella Clinica del Cittadino*, citizen’s clinical record). The TreC digital platform represents the citizen frontend to the local healthcare system and healthcare services. With the use of digital identity credentials (e.g. SPID identity), it can be accessed both via computers and via smartphones, allowing citizens to consult their Electronic Health Record, to manage their Personal Health Record and to access different public healthcare services (e.g. booking blood tests and medical exams, consulting medical prescriptions, paying medical tickets…) (for additional information see [20]).

The TreC platform incorporates various services through its middleware, that allow the ecosystem to be flexibly integrated with different devices, services and functionalities. Some basic services such as authentication and access control are implemented in order to allow patients to safely use the App and share information in a controlled way. Through the middleware, the patient’s App and the clinical web dashboard are connected and able to communicate (e.g. prescription of the App, televisit, chat, exchange of data). The correct functioning and the security of the technological infrastructure of TreC is constantly maintained and updated.

The TreC ecosystem also allows performing small tests before implementing a new service in the main citizen frontend. TreC Oculistica has been tested within a dedicated pre-production digital environment.

#### Patient’s App

**Fig 2:**
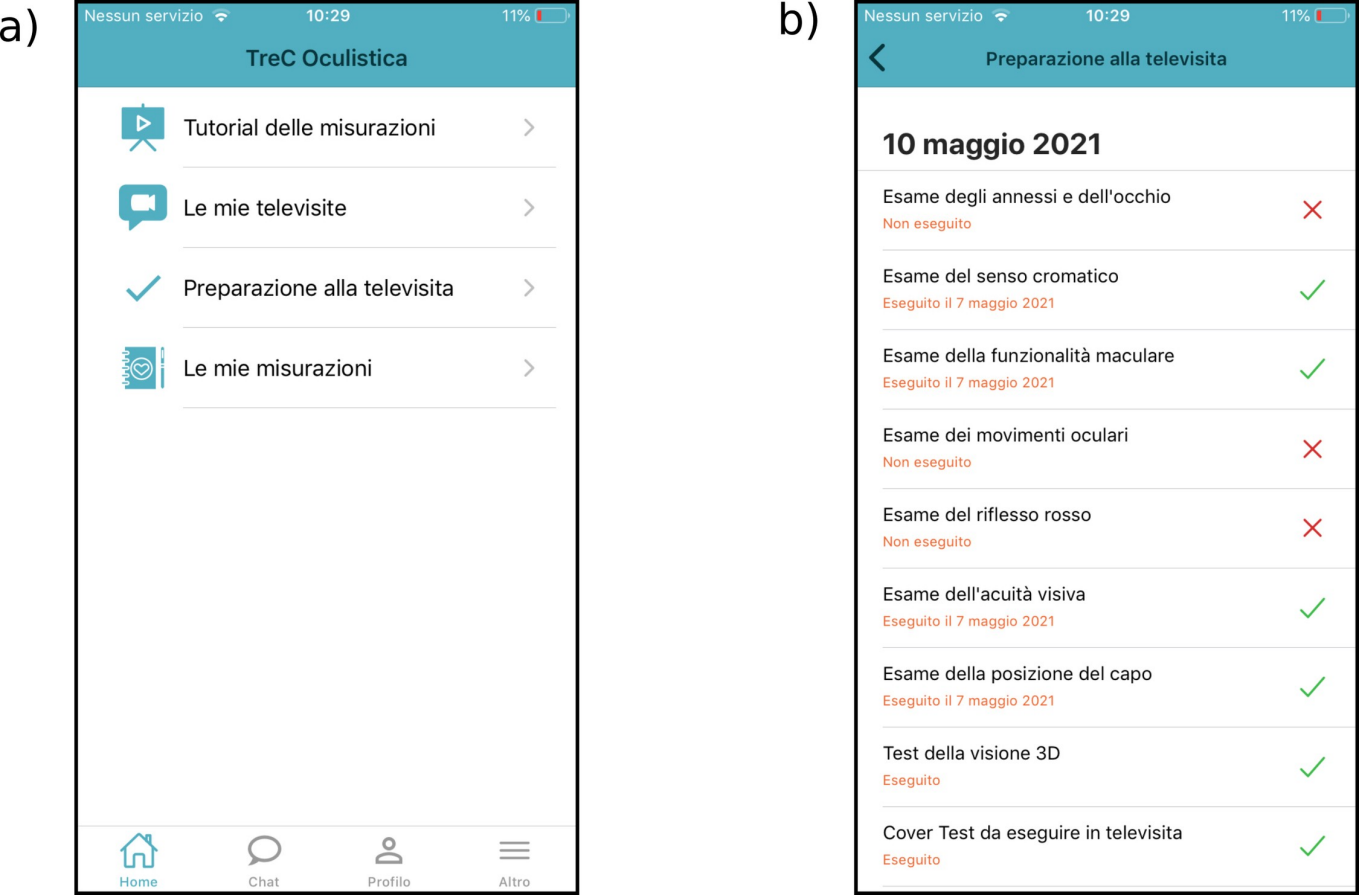
Examples of Patient’s App screens. **A: Patient’s App Home screen:** The main functionalities embedded in the platform are listed in the Home screen of patients’ mobile application. **B: Televisit preparation screen:** each required measurement is listed in the Televisit’s preparation screen. A red cross beside the measurement name indicates a non-completed task, a green tick indicates a completed task.

The TreC Oculistica patient’s App, available for both Android and iOS, can be accessed only after a healthcare practitioner prescription. The use of the app is legitimate within the boundaries of a doctor-patient relationship and its use is regulated by clinical purposes and within the legal frame imposed by the GDPR regulation. When downloading the App, the patient has to accept the Terms of Service and Privacy Policy and to complete the registration. Even though this initiative does not require participants’ informed consent, as it is a specific—albeit innovative—component of the standard healthcare service provision, we asked and collected a digital “consent for using the App” from each patient/caregiver. Then, only after his/her clinician prescribed the use of the App, he/she is allowed to access the functionalities of the App (Fig 2A). The App functionalities are:

- Measurements’ tutorials: in this area the patient can find one instruction file for each possible prescriptible measurement. Instructions are also provided for some exercises that will be performed real-time under the supervision of the clinician during the televisit.
- My televisits: when a new televisit appointment is scheduled patients receive a notification on their smartphone. Patients can visualize the appointment and answer the clinician video call in the *My televisits* area.
- Televisit preparation: Within the *televisit preparation* area, patients can find the list of the measurements required by the clinician. Each measurement is marked with a red cross until the data is inserted, then the red cross changes into a green tick (Fig 2B). By clicking on the name of each measurement, participants can access a form with a brief description of the measurement, the direct link to the tutorial explaining how to collect the information, the link to the suggested digital application (if present) and a dedicated space to enter the self-collected data.
- My measurements: this is the archive where the data about previous televisit appointments are stored.

In the bottom section of the App, patients can open the chat with the healthcare staff, visualize their own profile information (i.e. personal data), and access the helpdesk contacts, information on the App, and the Terms of Service.

#### Clinical dashboard for healthcare practitioners

The clinical dashboard is a web-based interface that allows ophthalmologists and orthoptists to record patients, prescribe the use of the App, schedule televisits and recommend preliminary measurements patients can collect at home, and to display data and measurements collected from the platform (i.e. patient App). The dashboard is accessible with valid credentials only to those healthcare practitioners that have been authorized by the platform administrator. The dashboard is integrated with the provincial health registry and allows to visualize the main personal data of each citizen in charge of the provincial healthcare organization (i.e. name, surname, date of birth, fiscal code).

**Fig 3:**
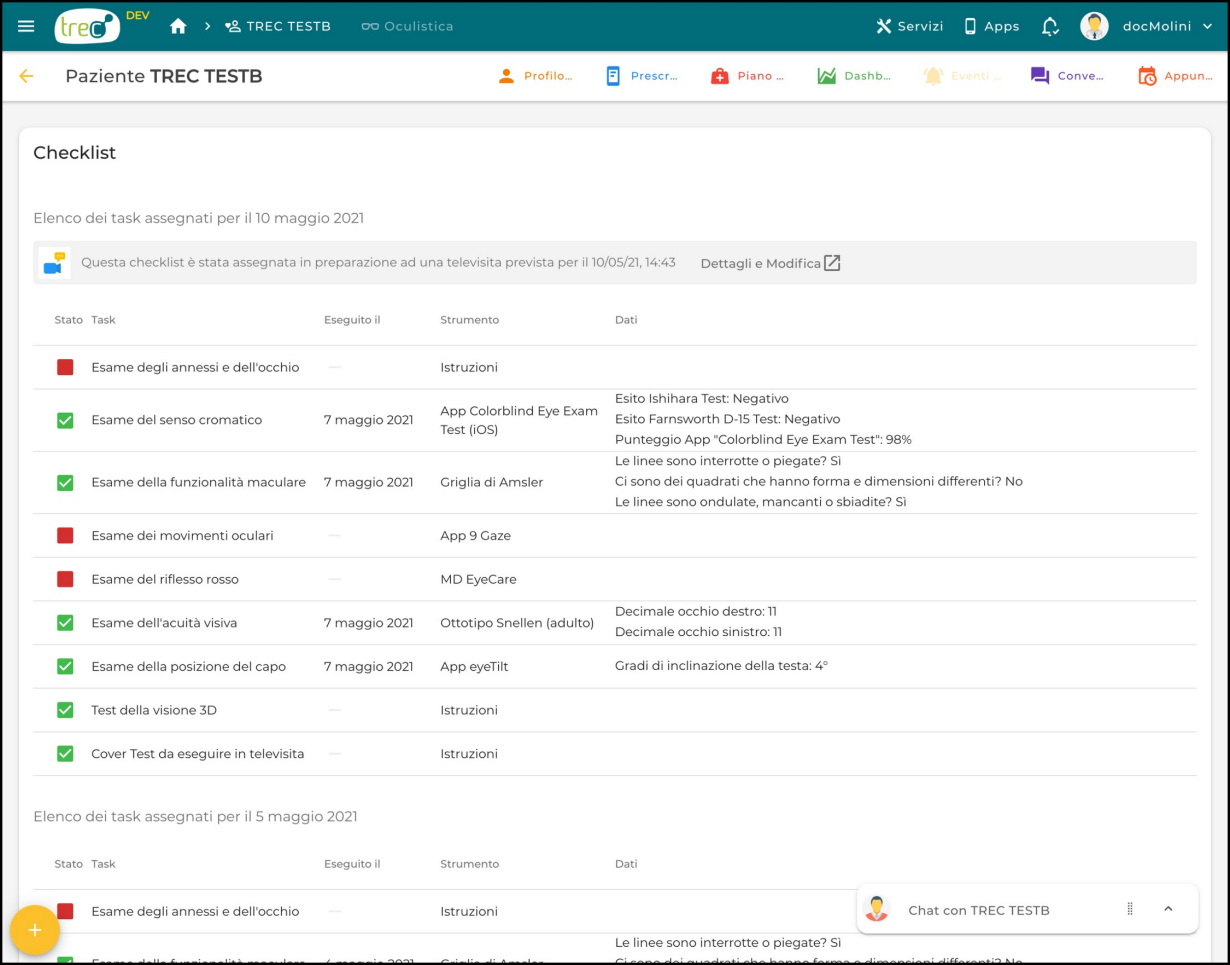
Clinical dashboard: For each scheduled televisit, the list of the requested measurements is displayed within a structured grid in the clinical dashboard. For each televisit, clinicians can visualize the state of the task (completed: green square; non-completed: red square), the name of the measurement, the data entry date, the suggested material, the patient’s data uploaded on the App.

Each enrolled patient has a dedicated clinical area that contains all the information the patient decides to share with the clinician. The measurements and the data related to a specific televisit, can be visualized in a structured matrix (Fig 3) avoiding misplacement or loss of information. Moreover, the dashboard allows to compare the data collected from the same patient at different times (i.e. different televisits). It also allows to personalize patients’ App by activating or deactivating the chat function, and by recommending different measurements according to patients’ eyesight deficit and ability. Clinicians can also communicate with their patients through the chat and program a televisit. While scheduling the televisit, clinicians can decide whether requiring some autonomous measurements to patients to better evaluate their eyesight condition. The measurements can be selected by ticking the corresponding voice from a checklist of measurements.

### Materials selection

The majority of ophthalmic visits, both first-visits and follow-up of pediatric or adult patients, require evaluating visual and visuo-motor functions, such as: visual acuity, eye motility, macular function, visual field and color vision (Table 1). These examinations can be easily self-administered by patients autonomously or with the help of a caregiver, using smartphone applications or printable materials.

**Table 1:** TreC Oculistica materials.

Examination	Name	Material
Visual acuity test	• HOTV optotype	• Printable test and tutorial
	• Snellen optotype	• Printable test and tutorial
	• LEA optotype	• Printable test and tutorial
	• App Snellen chart	• App + tutorial
	• App Tumbling E chart	• App + tutorial
Macular function test	• Amsler grid	• Printable test and tutorial
Visual apparatus and its annexes exam	• Photography of the eye	• Printable Tutorial
Eye movements exam	• App 9Gaze	• App + tutorial
Red reflex test	• MD Eye Care	• App + tutorial
Color vision test	• App Color Blind Test	• App + tutorial
Head posture test	• App Eye Tilt	• App + tutorial
Cover test to perform during the televisit	•	• Printable Tutorial
3D vision test	• Written instructions	• Printable Tutorial

The table summarizes the materials selected and available for self-measurements. The first column indicates the name of the supported examination, the second column reports the name of the selected materials, the third column reports the type of materials uploaded and indicated on the App (either printable materials or mobile applications).

The applications that have been identified within the TreC Oculistica App were selected considering the following criteria: ophthalmology-related medical applications, high sensitivity, ease of use/user friendliness, availability and free download.

The other materials loaded on the TreC Oculistica App were selected among those commonly used in the medical practice, where the visualization on printed paper or on a screen could be standardized based on different printers and digital devices.

To support patients and caregivers in the acquisition of measurements, we provided written tutorials illustrating the correct execution of each single measurement.

Mobile applications. *Visual Acuity Charts*. This App displays optically precise optotypes of eye charts to accurately measure distant visual acuity [23–25].

Supported OS: Android and iOS

Selected optotypes: Snellen for adults and Tumbling E for children

Test distance: 3 meters

Suggested notation: decimal

Test modes: manual or auto play

*9Gaze*. The App allows the creation of composite images aimed at monitoring eye motility and strabismus in the nine cardinal positions of gaze [26–28].

Supported OS: Android and iOS

*eyeTilt*. Digital goniometer aimed at measuring head tilts and turns using the mobile’s camera.

Supported OS: Android and iOS

Features: on-screen guide for placement of eyes and/or nose

*MD EyeCare*. The App performs the Red Reflex test instrumental in detecting opacities in the visual axis, such as a cataract or corneal abnormality, as well as abnormalities in the posterior segment, such as retinoblastoma or retinal detachment. It can also detect subtle differences in the red reflex between the eyes, indicating the presence of strabismus or refractive errors [29].

Supported OS: iOS

*Colorblind Eye Exam Test (iOS) and ColorBlind test (Android)*. Color blindness test aimed at assessing color deficiency, especially in the red and green tones.

#### Printable materials

*HOTV*, *ABCD-Vision (Anchorage*, *Alaska)*. paper-based HOTV crowding bar visual acuity test adapted to be self-administered remotely by patients [24, 30].

Test distance: 3 meters

*LEA Symbols*, *LEA-Test Ltd*. Visual acuity testing chart for pre-schooled children evaluation. The optotype is adherent to the World Health Organization (WHO) and the National Academy of Sciences Committee on Vision recommendations about optotype choice and arrangement on visual acuity testing charts [31, 32].

Test distance: 3 meters

*Snellen chart*. Snellen visual acuity testing chart for the evaluation of both adults and children [33].

Test distance: 3 meters

*Amsler grid*. A reticle with vertical and horizontal lines used to detect vision deficits resulting from macula (e.g. macular degeneration) or optic nerve (e.g. glaucoma) damages [34].

*Cover Test*. Instructions showing how to perform the cover test (cover-uncover and alternating) during the televisit. The test allows the detection of different forms of strabismus observing refixation movements.

*Hidden Image Stereograms*. Instructions and web-links were provided to investigate 3D vision by means of computer-generated Single Image Random Dot Stereograms (S.I.R.D.S) [35].

### The teleOphthalmology process

The process adopted to recruit each patient consisted in telephonically contacting the caregiver/patient, explaining the new system, asking him/her to download the App TreC Oculistica, plan a day for the televisit and enrolling the patient into the clinical dashboard by prescribing measurements and the televisit appointment.

Some days before the scheduled appointment, the caregiver/patient could ask for assistance in completing the measurements process using the chat, load the data collected and get ready for the televisit by reading the informative material. On the appointed day and time, the healthcare practitioner conducts the televisit. After the remote visit, the clinician would write a medical report and account for the televisit within the hospital IT system.

We identified three different scenarios that could be triggered by the televisit:

1. The televisit and the information collected are informative enough to state the patient’s sight condition and no additional in-presence evaluations are needed. The next follow-up visit is normally booked.
2. The televisit highlights the need for additional instrumental analysis. The patient can perform the examination in a local facility and then share the results with the hospital healthcare staff through the App. A new televisit is performed and the patient’s evaluation is completed.
3. The televisit shows the need for a face-to-face examination. An appointment is booked and the patient is visited in the clinic.

### Participants

Four clinicians (2 ophthalmologists and 2 orthoptists) were actively involved in testing the system. Each healthcare staff underwent an individual training session in order to familiarize with the system and be autonomous in its usage. Additionally, two manuals regarding the clinician web interface and the patient’s mobile App were provided for further support. A helpdesk service, available both for patients and clinicians, was activated for technical support and specific operative needs. After the training session, healthcare professionals were given access to the clinical web interface by the platform administrator.

**Fig 4:**
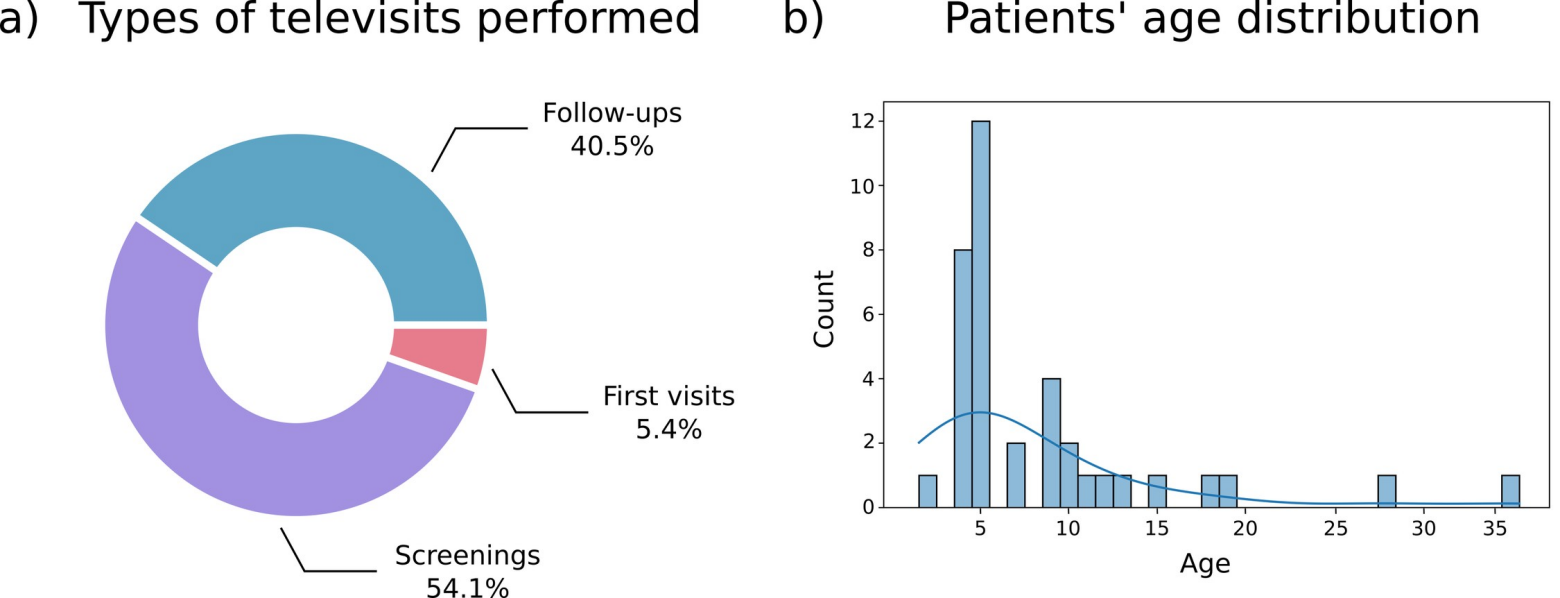
Televisits data. **A: Types of televisits performed:** the graph shows the percentage of the types of televisits performed. The orthoptic screening represents the majority of the televisits performed (54.1%), followed by follow-ups visits (40.5%). **B: Patients’ age distribution:** The graph represents the age distribution of the patients that underwent a televisit. Most of the represented patients are 5 years old childrens.

In the first stage of implementation (approx. three months), 54 patients were proposed a televisit, 37 patients (M = 26 F = 11; mean age: 8,5; median age: 5) (Fig 4) agreed to participate, downloaded the App and did a televisit. Because of the clinical specialization of the healthcare practitioners involved, the great majority of the patients were accessing the pediatric ophthalmology department and were identified within the waiting list. The main diagnosis is reported in Table 2.

**Table 2:** General aim of televisits.

Pediatric Ophthalmology televisits (type of visit and main diagnosis)	Number of cases
Conjunctival neoformation follow-up	1
Post-operative strabismus check-up	7
Follow-up in patient with mild microphthalmia with polar cataracts	1
Follow-up in patient with pigmentary retinopathy (Mud-splattered fundus)	1
Follow-up in patient with accommodative strabismus	1
Follow-up in patient with unilateral cataract	1
Follow-up in patient with plagiocephaly (and fourth nerve palsy)	1
Visual acuity test (first visit)	2
**Orthoptic televisits**	**Number of cases**
Orthoptic screening	20
Follow-up: visual acuity test in strabismus	1
Follow-up: diplopia evaluation in strabismus	1

The table reports the main diagnosis of each televisited patient divided by the ophthalmic and orthoptic domain and the count of the televisited cases. Overall, post-operative strabismus check and orthoptic screening represent more than half of the televisited cases.

The televisits were performed both with residents in the Province of Trento (N = 35) and with patients living outside the Region (N = 2). Aiming at testing mainly the use of the platform and of telemedicine within the Ophthalmic Unit, a conservative patients selection strategy was adopted discarding those with a complex clinical picture. As a result, none of the televisited patients needed to access an in-person visit. Patients involved in the testing phase reported to be overall satisfied.

## Discussion

In this paper we described the genesis and the evolution of an innovative approach to teleophthalmology that took place during the COVID-19 outbreak in the Province of Trento. To our best knowledge this is the first structured attempt in the Italian healthcare system to design and implement a technological solution for teleophthalmology to cope with the new challenges the pandemic highlighted. The COVID-19 unprecedent circumstances have encouraged the adoption of telemedicine in different medical fields exploring different approaches. Similarly to the approach adopted in the present study, in the neurology field the possibility to perform some evaluation at a distance has been tested. With the use of mobile phone’s applications, wearable devices, e.g. accelerometers, or the remote administration of clinical tests, clinicians were able to successfully assess patient’s condition reducing the hospitalization and improving patients’ quality of life [36–39].The spread like wild fire of telemedicine is a significant event that need to be seriously examined. The healthcare system and its main actors (patients and clinicians) seem to be now ready for a transition from a “traditional” provision of services to a digital health. Another innovative aspect of this experience is the emphasis given on the empowerment of patients and their involvement in managing their own health, as well as in the visit’s process. This is not a new concept for many clinical fields (e.g. management and telemonitoring of chronic diseases) [40–42], but so far it has not been extensively explored in the ophthalmic scenario. Usually, information is collected by technician and trained personnel, and then shared with specialists through a teleconsultation (the so-called “provider-collected information”). Here, in light of the social-distancing regulation, we designed a context where patients are proactively involved in the process of collecting information and parameters in advance to the televisit appointment, providing a safe and reliable communication channel. We built a platform that can be easily used by patients/caregivers and clinicians, and we provided indications and support to reduce the digital gap that is still present among the population. At the same time, this initiative has been promoting a novel relation between patient and healthcare staff, with a co-construction and mutual collaboration in collecting clinically relevant data within a telemedicine approach. A co-design approach involving a public stakeholder, such as the Competence Center on Digital Health TrentinoSalute4.0, and medical practitioners favored the perception of this technology as a legitimate channel to communicate and exchange information within the patient-doctor relationship.

The introduction of a new digital solution can be extremely challenging. In the following paragraphs we will provide an overview of both pitfalls and limitations encountered, lessons learnt and future steps.

### Pitfalls and limitations

#### Digital divide

Even if designed to be intuitive and user friendly, technology is not accessible to everybody. In a recent paper, Scanzera et al. [43] highlighted that the COVID-19 pandemic exacerbated digital divide and inequities in accessing telehealth services. The authors showed this is not only an age-related issue, but it also affects low-income segments of the general population, which are more likely to experience difficulties in accessing digital devices (i.e. smartphones, computers, tablets) and services (e.g. internet connection).

Together with all the above-mentioned considerations, we have also found that language might be a limitation in the adoption of a digital solution. The TreC Oculistica interface is in Italian; nevertheless, in a growing multicultural society, digital technologies should be accessible also to non-native speakers. This of course has some other implications, such as increasing costs in developing digital solutions and time constraints in the release of the system.

Moreover, some people are more prone than others in trusting new technologies [7]. There is always a psychological component that is interfering in the full adoption of a new digital solution, and this is true both for patients and clinicians. Out of 54 patients contacted, only 37 accepted to perform televisit. Further studies investigating the acceptability of telemedicine solutions and exploring the dynamics of the changes induced by digital innovation in the healthcare system are needed. We can just hypothesize that the physical distance characterizing telemedicine services, and the novelty of digital health solutions, might have somehow impacted on the availability of specific subset of patients in approaching this new opportunity. Among the families that did not want to try the televisit three main groups emerged: i) families that initially agreed in participating and then did not show up at the scheduled appointment; ii) families that did not want to try because found the protocol too complicated; iii) families that doubted on their child’s collaboration. On the other hand, it is worth noting that few families (N = 5) even when the Covid-19 safety measurements would have allowed to perform a face-to-face visit, refused to perform a in presence visit and rather preferred to continue with televisits instead.

#### System limitations

The testing of TreC Oculistica has been extremely useful in understanding limitations and ameliorations to implement in future releases.

One of the first considerations that emerged was that the evaluation of some patients at a distance would have been easier if patients could rely on a bigger screen. A web version of the App would respond to this need allowing patients to undergo ophthalmic televisits more comfortably. Nevertheless, it has to be considered that some smartphones are equipped with high resolution webcams that convey a better image resolution compared to standard PC webcams. The acceptable balance between image resolution and screen size should be established for each televisit according to the available devices.

Moreover, in the material selection we tried to select free-of-charge medical mobile applications, as we did not want this initiative to require additional economical effort from patients. However, it is possible that some of these applications will not be free-of-charge in the future. Currently, the Ophthalmic Unit is considering alternative materials (e.g. printable materials or web links) that could substitute the use of paid Apps. We have also found that visual instructions (i.e. video-tutorial), instead of written instructions, would be much more effective and enjoyable for App users. We are currently realizing some video tutorials to upload on the App in the Measurement’s tutorial section.

The integration of the TreC Oculistica system with the hospital IT system (i.e. medical reporting) would also optimize and encourage the use of the system, as clinicians could complete administrative (e.g. medical report) and clinical (e.g. televisit) procedures accessing a single interface.

In addition, in few areas of the Region the internet connection quality is not optimal, causing difficulties in conducting televisits. Overall, even if on a small scale, this test is useful in identifying practical obstacles that, if not address, could interfere with the success of a virtuous initiative.

#### Qualitative assessment of reliability in self-collected parameters

As argued by Saleem et al. [44], there is no factual proof that could validate the reliability of self-collected parameters. Thus, how can we be sure that the values patients inserted in the TreC App reflect their real vision condition? Unfortunately, we are not able to claim that these parameters are indeed correct; however, the literature shows that there is a good reliability in self-collected vs. professional-collected information in different fields [45–49]. To avoid possible inaccuracies, our clinicians adopted a conservative approach during the first test of the TreC Oculistica system. For known-patients, they were able to compare previous data to self-measurements; for new patients undergoing a screening evaluation, a subsequent in-person visit was scheduled after the end of the pandemic emergency so that clinicians could monitor and compare the two datasets. To better assess the reliability of self-collected information, clinicians started to ask patients how confident they were while measuring a certain parameter using a Likert scale (values ranging from 1 –no confidence at all, to 4 –very confident). Even though our sample size is not statistically significant yet, we have noticed that the introduction of this simple parameter can really improve our assessment. In any case, future research comparing self-collected information and provider-collected data could be useful in view of validating specific tele-ophthalmology protocols.

#### Ethical issues

When considering the transmission of sensible data, privacy issues and data management are extremely important [50]. The COVID-19 emergency promoted a higher level of tolerance when using platforms not specifically designed nor apt for clinical use (e.g. Gmail) to provide healthcare assistance in spite of the pandemic and its limitations. The TreC platform is GDPR compliant and ensures high safety and reliability standards. As the scope and purpose of data processing for this initiative is linked to the provision of a standard—albeit innovative—healthcare service, no need is foreseen for collecting an informed consent. However, for sake of transparency and to improve users’ understanding of this specific service, patients/caregivers that want to use the App need to accept an information sheet, describing data treatment guidelines and protocols. Moreover, for each suggested App a specific cross-check of privacy policies was performed, discarding those that did not have one.

### Lessons learnt and future steps

Despite the tragic outbreak period, the COVID-19 emergency provided the public health sector with the opportunity to re-think the health organization and paved the way for testing and implementing new digital services. If, as reported by Gioia and Salducci [51], in 2019 there was not a diffuse use of telemedicine in Europe, we acknowledge that different and spontaneous telemedicine initiatives scattered during the COVID-19 outbreak [51]. This suggests that healthcare systems and societies are getting ready to embrace new digital services.

In the past few years, the Province of Trento (Italy) implemented different digital solutions to improve the quality of services provided by the local healthcare [51–53]. In this context, the Competence Centre on Digital Health TrentinoSalute4.0, plays a pivotal role in supporting the development and adoption of digital solutions in the healthcare system [54].

Overall, the first attempt in approaching teleophthalmology has been positive. The co-designed system has been well accepted by patients and clinicians as it is easy to use and intuitive. It represented a good help in monitoring and supporting outpatients despite social-distancing limitations. Telemedicine, when possible, is convenient not only for the healthcare system but also for patients, that can avoid transportations to reach central hospitals, saving time and money (i.e. gas and parking) [7]. We mainly focused on pediatric ophthalmology, a sub-specialty whose characteristics are particularly suited to telemedicine. Typically, the caregiver of a pediatric patient is around 30–40 years old, meaning that their digital knowledge (e.g. how to use a smartphone or a computer) is likely to be higher compared to the elderly population. We have found that children are really cooperative when collecting the information asked by clinicians at home. In their familiar environment young patients are usually more relaxed than in clinical settings [55]; the measurement itself is perceived as a game, and often the whole family is involved in collecting the required parameters.

Improving patients’ satisfaction toward a service is not always enough to amend a structural organization. Evaluating the economic impact of digital services is necessary to understand the sustainability of an innovation. New evidence is produced about the economic impact of digital innovations, showing cost savings for the healthcare system [56]. The introduction of telemedicine impacts also the Operative Units’ organization. Clinicians need to get proper training to feel confident in using technological solutions, and televisit needs to be structurally introduced in the daily scheduled activities [44]. This would also allow the healthcare staff to experiment forms of smart working, optimizing the use of the clinic space and improving working conditions.

Smartphone applications are an interesting tool in the teleophthalmology scenario, nevertheless how they could be used in the clinical practice is an open question [57]. Should the ophthalmology applications be certified as medical devices in order to be extensively used in the clinical practice? How can their use be addressed in the privacy scenario? Should a public health system, such the Italian one, invest in the creation of mobile phone applications for clinical purposes? The constantly evolving scenario of digital health will impose a common and shared vision of limits and possibilities of telemedicine. It is undisputed that several initiatives experimented in different parts of the world identified some positive and desirable prospective for the future despite cultural geographical differences. Investing in telemedicine seems to be a win-win scenario; however, in order to reduce the digital divide and allow more patient to access digital services with the help of an intermediary (e.g. nurses or orthoptists), a hybrid model could be considered, combining instrumental examination that can be performed by non-specialized technicians in peripheral offices and real-time televisits [8, 43]. This is the scenario we plan to invest in, where ophthalmic devices, digitally connected with a central reading center, will be located in nursing facilities around the Region. Patients whose conditions do not require an in-presence-visit, will find support in undergoing instrumental examinations and accessing telemedicine services.

## Conclusions

The process described herein represents a valuable example of health digitalization during COVID-19 outbreak. Even if this experience has been implemented on a limited number of patients (so far), the proficient contribution of the group of pediatric ophthalmologists and orthoptists has led to a promising protocol which is now ready for scale up.

Despite the fact that the COVID-19 pandemic sadly represented the incentive, arose by a necessity, to walk out of the comfort zone of known clinical procedures, this context has promoted the adoption of a different and challenging approach in teleophthalmology, which proved to be potentially solid.
